# The Evolution of a Journal

**DOI:** 10.1155/S1064744996000610

**Published:** 1996

**Authors:** Sebastian Faro


					Infectious Diseases in Obstetrics and Gynecology 4:311 (1996)
(C) 1997 Wiley-Liss, Inc.
The Evolution of a Journal
The journal Infectious Diseases in Obstetrics and Gynecology is going through an evo-
lution that began four years ago. Initially, we published clinical, basic science, and
review articles. Recently two new sections were added; Images, which presents
color photographs of interesting clinical cases, and the Antibiotic Symposium,
which brings information on new antimicrobials as well as updates on existing
agents. Thus, the journal has become a comprehensive vehicle to desseminate
information on infections in obstetrics and gynecology.
In keeping with progressive developments, in this issue the journal is publish-
ing the abstracts of the annual meeting of the Infectious Diseases Society for
Obstetrics and Gynecology. It is a great pleasure and an honor to publish their
abstracts annually in our capacity as the official journal of this society.
The journal continues to grow into a more comprehensive publication while
striving to be listed in Index 31edicus. In order to achieve this goal, the journal will
continue to improve while trying to keep a consistent publication schedule. Ad-
ditionally, the journal will need to increase the number of manuscripts published
in each issue. This will only occur if individuals with an interest in infectious
diseases submit manuscripts for review. am happy to report that colleagues from
across the Atlantic and Pacific oceans are increasing their contributions. It is only
with the support of the readership and investigators that this journal can develop
the stature that will enable it to succeed and become listed in Index 3Iedicus.
Sebastian Faro, MD, PhD
Editor-in-Chief
Infectious Diseases in Obstetrics and Gynecology
Editorial

				

